# The effects of short-term high-fat feeding on exercise capacity: multi-tissue transcriptome changes by RNA sequencing analysis

**DOI:** 10.1186/s12944-017-0424-7

**Published:** 2017-02-02

**Authors:** Ya Xiao, Wanshan Wang, Liguo Chen, Jieyu Chen, Pingping Jiang, Xiuqiong Fu, Xiaoli Nie, Hiuyee Kwan, Yanyan Liu, Xiaoshan Zhao

**Affiliations:** 1 Department of Traditional Chinese Medicine, School of Medicine, Jinan University, Guangzhou, China; 2 School of Traditional Chinese Medicine, Southern Medical University, Guangzhou, China; 3 Experimental Animal Center, Southern Medical University, Guangzhou, China; 4 School of Chinese Medicine, Hong Kong Baptist University, Hong Kong, China

**Keywords:** RNA Sequencing, High-fat feeding, Exercise capacity, Multi-tissue, Short-term

## Abstract

**Background:**

The effects of short-term high fat diets on physiology are elusive and the molecular changes following fat overconsumption remain largely unknown. In this study, we aimed to evaluate exercise capacity in mice fed with a high fat diet (HFD) for 3 days and investigate the molecular mechanisms in the early response to high-fat feeding.

**Methods:**

Exercise capacity was assessed by weight-loaded swimming test in mice fed a control diet (10 kcal% fat) or a HFD (60 kcal% fat) for 3 days. Global gene expression of ten important tissues (brain, heart, liver, spleen, lung, kidney, stomach, duodenum, skeletal muscle and blood) was analyzed using RNA Sequencing.

**Results:**

A HFD for just 3 days can induce 71% decrease of exercise performance prior to substantial weight gain (*P* <0.01). Principle component analysis revealed that differential gene expression patterns existed in the ten tissues. Out of which, the brain, spleen and lung were demonstrated to have more pronounced transcriptional changes than other tissues. Biological process analysis for differentially expressed genes in the brain, spleen and lung showed that dysregulation of peripheral and central immune response had been implicated in the early stage of HFD exposure. Neurotransmission related genes and circulatory system process related genes were significantly down-regulated in the brain and lung, respectively.

**Conclusions:**

Our findings provide new insights for the deleterious effects of high-fat feeding, especially revealing that the lung maybe as a new important target attacked by short-term high-fat feeding.

**Electronic supplementary material:**

The online version of this article (doi:10.1186/s12944-017-0424-7) contains supplementary material, which is available to authorized users.

## Background

High-fat and high-calorie diets along with a low physical activity lifestyle have contributed to the onset or development of type 2 diabetes, metabolic syndrome and cardiovascular disease [1]. There has been conflicting results on whether consumption of a high fat diet (HFD) is detrimental or beneficial for endurance performance. Studies in rats have demonstrated a beneficial effect of a fat-rich diet on exercise capacity via increasing the ability to oxidize fat and concomitantly sparing glycogen content [2–4]. In contrast, Murray et al. [5] reported that 9 days of high-fat feeding impaired energy production and physical performance associated with respiratory uncoupling in skeletal muscle mitochondria. In the present study, we aimed to evaluate exercise capacity in mice fed with a HFD for 3 days and investigate the molecular mechanisms in the early response to high-fat feeding.

Advances in genomic technologies may help to reveal the early molecular changes by enabling simultaneous analysis of thousands of genes in response to a HFD. The serial analysis of gene expression strategy identified 12 transcripts of hypothalamus which regulated by food intake in mice at 3 h after high-fat meal ingestion [6]. The transcriptomic analysis of duodenum mucosa after high-fat meal ingestion in C57BL/6 J mice found substantial changes of genes related to lipid metabolism [7]. Microarray analysis showed markedly changes of numerous genes involved in various biological processes including morphogenesis, fatty acid catabolism and amino acid metabolism following 3 days of high-fat feeding in the skeletal muscle of C57BL/6 J mice [8]. cDNA microarrays analysis of mRNA expression showed down-regulation of genes related to fatty acid biosynthesis in the liver of one week HFD-fed BALB/c mice [9].

However, no study to date has simultaneously analyzed the systemic gene expression profile of multi-tissues in response to short-term HFD and it remains unknown that which tissue has the most pronounced changes of gene expression profile in the early stage after high-fat feeding. Recently, RNA sequencing (RNA-seq), as an attractive alternative to microarrays for transcriptome analysis, provides major advances in robustness, comparability and richness of expression profiling data [10]. Thus we utilized RNA-seq to investigate gene expression profile of ten tissues (brain, heart, liver, spleen, lung, kidney, stomach, duodenum, skeletal muscle and blood) in C57BL/6 J mice with 3 days of high-fat feeding, which may contribute to the understanding of molecular mechanisms of changes in exercise performance induced by short-term HFD.

## Methods

### Animals and study protocol

Animal experiments were approved by the Animal Care and Use Committee of Southern Medical University (Approval No.2013027). The methods were carried out in accordance with the approved guidelines. Forty male C57BL/6 J mice at the age of 8 weeks were obtained from Laboratory Animal Center of Southern Medical University (Approval No. SCXK (Yue) 2011–0015). All the animals were maintained in a temperature-controlled room (22–25 °C; 35–55% humidity) with a twelve-hour light/dark cycle. Mice were randomly divided into two groups, where 20 mice were fed a control diet (CD, D12450B, 10 kcal% fat) and 20 mice were fed a high fat diet (HFD, D12492, 60 kcal% fat) for 3 days. Mice were allowed free access to food and water. The changes of body weight were observed after 3 days.

### Assessment of exercise capacity

A weight-loaded swimming test has been commonly used for assessment of exercise capacity in murine [11, 12]. After 3 days, 10 mice were taken out from each group for swimming exercise performance test which was conducted as previously described with some modifications [13]. The mice were not fasted and were loaded the constant weight (1.5 g tin wire, attached to the tail). The mice were dropped individually into a swimming pool (30 cm high, 25 cm in diameter) filled with water at 25 ± 1 °C. It was considered that the mice were exhausted when they failed to return to the surface of water within a 10 s period. The swimming time to exhaustion was used as the index of exercise capacity.

### Biochemical assays

After 3 days of feeding, the remaining 10 mice in each group were anesthetized with sodium pentobarbital (75 mg/kg, ip) following a 6 h fasting period. The blood samples were collected by removing the left eyeball of the mice and rapidly centrifuged at 1000 *g* at 4 °C for 10 min. Plasma levels of glucose, triglycerides, total cholesterol, low-density lipoprotein-cholesterol (LDL-C), high-density lipoprotein-cholesterol (HDL-C), free fatty acids (FFAs), apolipoprotein E (ApoE), C-reactive protein (CRP), superoxide dismutase (SOD), homocysteine (HCY), alanine aminotransferase (ALT), aspartate aminotransferase (AST), alkaline phosphatase (ALP), total protein (TP), albumin (ALB), globulin (GLB), ALB/GLB, total bilirubin (TBIL), direct bilirubin (DBIL), indirect bilirubin (IBIL), total bile acid (TBA), uric acid (UA), Creatinine (Cr), urea, Cystatin C (CysC), creatine kinase (CK), lactate dehydrogenase (LDH), A-hydroxybutyric acid dehydrogenase (HBDH), potassium (K), sodium (Na), chlorine (Cl) and calcium (Ca) were measured using a multifunctional biochemistry analyzer (Olympus AU2700, Tokyo, Japan). Statistical analyses for biochemical assays and assessment of exercise capacity were conducted using SPSS (version 19.0) for Windows. The data are reported as mean ± standard error of the mean (SEM). Differences between the compared groups were analyzed by Student’s *t* test. A *P* value less than 0.05 was considered to be statistically significant.

### Tissue Processing and RNA Isolation

Five mice of each group were selected randomly from the mice which did not perform weight-loaded swimming test for RNA sequencing. Tissue samples included the whole brain and heart, liver, spleen, lung, kidney, stomach, duodenum, skeletal muscle and blood. The samples were dissected and immediately immersed in RNA later solution (Ambion, California, USA). All samples were stored at – 80 °C before processing. Total RNA was extracted from all samples using Trizol reagent (Invitrogen, Carlsbad, CA). The RNA concentration was quantified using a spectrophotometer (NanoDrop echnologies, Wilmington, DE) and the integrity was evaluated by the Agilent Bioanalyzer 2100 (Agilent, Santa Clara, CA).

### RNA sequencing and gene expression analysis

In the CD and HFD group, fixed quantities of RNA of five samples from the same kind of tissue were combined into a single sample. The cDNA library was conducted by Illumina Tru-Seq RNA Sample Prep Kits (Illumina, San Diego, CA) with Ribosomal RNA depletion following manufacturer’s instructions. Samples were sequenced for 50 bp single read using the HiSeq2000 platform. Before alignment, reads with a low quality and adapters were screened by FastQC and removed. The remaining reads were mapped to the mice reference genome (UCSC mm10) with TopHat v2.0.9. The maximum number allowed for mismatch mapping was 2. Reads Per Kilobase of exon model per Million mapped reads (RPKM) was calculated to express the mRNA abundances. Analysis of differential expression was performed using edgeR, which could be used even with the most minimal levels of replication [14]. The read counts per gene were normalized to counts per million (CPM). CPM values were utilized for differential expression analysis, whereas RPKM values were used for principle component analysis (PCA) with the GeneSpring Gx 12.0 software (Agilent Technologies, Palo Alto, CA).

### Biological process analysis

Molecule annotation system (MAS) is a set of web tools for function annotation based on integration of various public resources such as Gene Ontology, KEGG, BioCarta, GenMapp, UniGene, OMIM and more [15]. Biological process analysis for differentially expressed genes (DEGs) was performed using the CapitalBio MAS 3.0 software (CapitalBio Corporation, Beijing, China). Absolute fold change >2 with *P* < 0.05 was considered statistically significant in the RNA-seq analysis.

### Real-time quantitative RT-PCR verification of RNA-seq data

To further confirm the findings from the RNA-seq analysis, we selectively examined 22 genes expression (8 genes in the brain, 9 genes in the spleen and 5 genes in the lung) using real-time quantitative RT-PCR (qRT-PCR) method. Five samples from the same kind of tissue of purified RNA in each group were used for qRT-PCR. Total RNA from the samples was first reverse- transcribed into cDNA templates with the PrimeScriptTM RT reagent Kit (TaKaRa, Otsu, Japan) according to the manufacturer’s instruction. PCR was run on a ABI 7500 Real-Time PCR System (Applied Biosystems, Inc., Foster City, CA, USA) using the SYBR Premix Ex TaqTM II (Otsu-Shi, Shiga, Japan). The reaction volume was 20 μL and the PCR conditions were as follows: 30 s. at 95 °C, 40 cycles of 5 s. at 95 °C and 34 s. at 60 °C, followed by a melting curve analysis step. Every sample was measured in duplicate, and relative quantification was determined by the comparative Ct method (2-ΔΔCT). β-actin was used as a housekeeping gene to normalize the expression data. The primers used for gene validation are listed in Additional file 1: Table S1.

## Results

### Body weight and blood plasma metabolites

As shown in Table 1, initial and final body weights showed no significantly differences among the groups. HFD feeding tended to increase weight gain, but this effect failed to reach statistical significance (*P* = 0.094). Plasma glucose levels were 67% higher in HFD-fed mice than CD-fed mice (*P <* 0.001). Although no differences were seen in plasma LDL-C, FFA and ApoE, the cholesterol level was significantly greater in the HFD-fed mice (*P* < 0.001). The increase in HDL-C (*P* < 0.001) and decrease in plasma triglycerides (*P* = 0.005) were also observed in HFD-fed mice. Plasma levels of CRP, SOD, HCY, ALT, AST, ALP, TP, ALB, GLB, ALB/GLB, TBIL, DBIL, IBIL, TBA, UA, Cr, Urea, CysC, LDH, HBDH, K and Ca were unchanged between the CD and HFD groups except for the CK, Na and Cl (Additional file 2: Table S2).

**Table 1:** Body weight and plasma biochemical parameters of CD-fed and HFD-fed mice

Parameter	CD	HFD	*P* value
Initial body weight (g)	22.81 ± 0.29	22.84 ± 0.31	0.950
Final body weight (g)	22.64 ± 0.40	23.45 ± 0.26	0.094
Plasma glucose (mmol/L)	4.71 ± 0.42	7.89 ± 0.31	<.001
Plasma triglycerides (mmol/L)	0.46 ± 0.03	0.36 ± 0.01	0.005
Plasma cholesterol (mmol/L)	2.16 ± 0.08	3.25 ± 0.08	<.001
Plasma LDL-C (mmol/L)	0.13 ± 0.02	0.19 ± 0.04	0.233
Plasma HDL-C (mmol/L)	1.55 ± 0.05	2.30 ± 0.04	<.001
Plasma FFA (mmol/L)	0.85 ± 0.04	0.88 ± 0.04	0.611
Plasma ApoE (mg/L)	16.34 ± 3.24	21.30 ± 2.57	0.254

Values are expressed as means ± SEM. CD, control diet; *HFD* high fat diet

### Exercise capacity in a weight-loaded swimming test

**Fig. 1:**
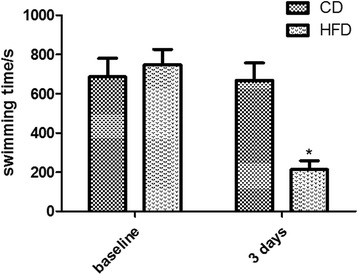
Effects of high-fat diet feeding on exercise capacity in C57BL/6 J mice (CD, control diet; HFD, high fat diet; *P < 0.01 vs. CD-fed mice). Values are expressed as means ± SEM

The swimming time indicated the exercise capacity. Both groups of mice swam the same mean time at baseline (CD = 687.3 ± 93.9 s, HFD = 669.0 ± 87.8 s). After 3 days of feeding, CD-fed mice maintained a similar swimming time of 748.9 ± 77.4 s, whereas HFD-fed mice swam 213.7 ± 44.4 s on average, 71% less far than the CD-fed mice (*P* <0.001) (Fig. 1).

### Summary of sequencing data and global gene expression profiles

A range of 28.1 to 67.4 million raw reads were generated among samples. After removing reads with a low quality, an average of 32.3 million clean reads per sample was obtained (range, 21.3 to 51.5 million reads). Approximately 98.64% of clean reads per sample were mapped to the mice reference genome among samples (Table 2). Totally 33151 unique genes among all samples was detected. To assess the effect of sequencing depth on RNA-seq data, we conducted sequencing saturation analysis. In the beginning of the RNA-seq, with increase of the counts of reads, the number of identified genes in each tissue was increasing. However, when the counts of reads rose to approximately 30 million, the growth rate of identified genes flattened which indicated that the number of identified genes tended to saturation.

**Table 2:** Summary of sequence statistics

Sample	Total number of raw reads	Total number of clean reads	Mapped reads	Mapping ratio (%)
C-blood	38,700,424	30,614,380	30,228,909	98.74%
C-brain	52,264,883	40,188,570	39,152,260	97.42%
C-duodenum	59,741,001	45,938,467	45,365,742	98.75%
C-heart	30,948,992	23,609,651	23,392,762	99.08%
C-kidney	52,803,870	40,421,377	40,024,878	99.02%
C-liver	36,944,726	28,422,843	28,121,458	98.94%
C-lung	28,118,618	21,811,801	21,603,740	99.05%
C-muscle	38,907,555	29,713,640	29,393,124	98.92%
C-spleen	46,764,891	36,109,723	34,944,640	96.77%
C-stomach	45,416,694	34,795,951	34,206,646	98.31%
H-blood	58,119,027	46,530,915	46,004,745	98.87%
H-brain	29,603,099	22,773,050	22,369,746	98.23%
H-duodenum	49,387,591	37,878,983	37,445,186	98.85%
H-heart	38,642,015	29,564,572	29,298,974	99.10%
H-liver	67,430,163	51,556,269	51,032,926	98.98%
H-lung	32,460,683	25,169,611	24,916,857	99.00%
H-muscle	30,898,190	23,377,884	23,142,897	98.99%
H-spleen	31,165,867	24,237,199	23,688,869	97.74%
H-stomach	27,849,155	21,301,788	21,072,130	98.92%
H-kidney	43,190,796	33,268,647	32,945,285	99.03%

*C* control diet group, *H* high fat diet group

**Fig. 2:**
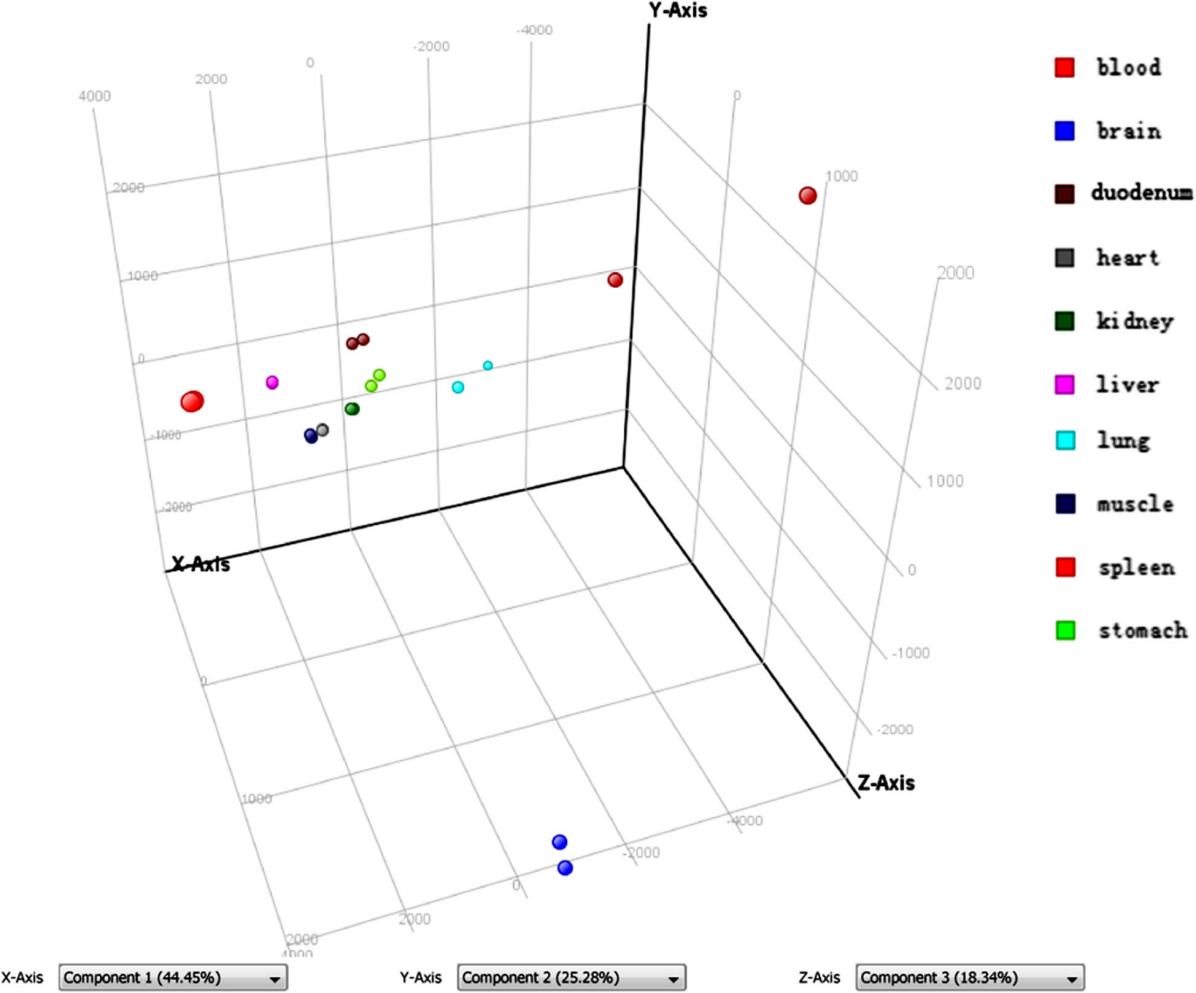
Principle component analysis of ten tissues in CD-fed and HFD-fed mice (CD, control diet; HFD, high fat diet). PCA analysis was conducted using the GeneSpring Gx 12.0 software. Each sphere represents an individual sample. The sphere representing *liver*, *heart*, *kidney*, skeletal muscle and *blood* in the two groups overlapped

To investigate the global gene expression profiles of ten tissues and identify the tissue with the most pronounced transcriptional changes after short-term high-fat feeding, we performed PCA on all samples (Fig. 2). The results showed differential gene expression patterns in the ten tissues. Each sphere represented an individual sample. The sphere representing liver, heart, kidney, skeletal muscle and blood in CD and HFD group overlapped, indicating that gene expression patterns of HFD-fed mice in the five tissues were almost not changed. The sphere representing stomach and duodenum in CD and HFD group were closely to each other, indicating that gene expression patterns of CD-fed and HFD-fed mice in the two tissues were nearly similar. Gene expression patterns of brain, spleen and lung in HFD group showed substantial differences as compared with CD group.

### Genes and the related biological processes altered in the brain of HFD-fed mice

**Fig. 3:**
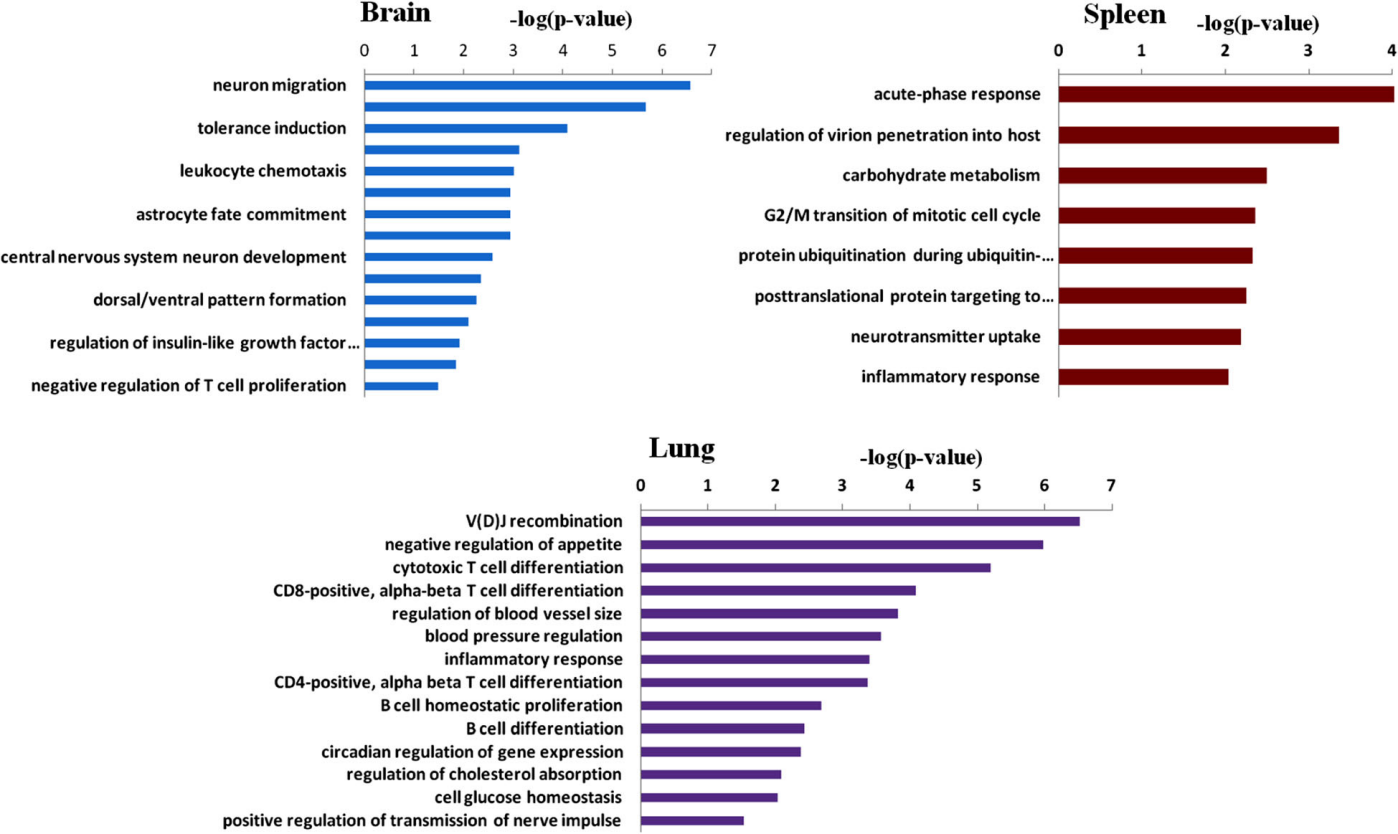
Enriched biological process significantly altered in HFD-fed mice compared to CD-fed mice in the *brain*, *spleen* and *lung *(CD, control diet; HFD, high fat diet)

According to the results of PCA, we found that the brain, spleen and lung had more pronounced transcriptional changes than other tissues following 3 days of HFD intervention. Consequently, we focused on the analysis of the genes and related biological processes altered in the brain, spleen and lung of HFD-fed mice. We found 145 DEGs in the brain, of which less than half of the genes were annotated with known function from the Ensembl database (Table 3). To gain insight into the possible biologic functions of the genes affected by high-fat feeding, enrichment analysis of Gene Ontology for the DEGs was conducted. After 3 days of HFD exposure, the overrepresented biological processes in the brain were mainly enriched in neurological system process and immune response (Fig. 3). In the neurological system process related group, LIM homeobox transcription factor 1 beta (Lmx1b), and NK2 homeobox 1(Nkx2-1), which involved in neuron migration and development, were down-regulated to 8.11-fold and 10.56-fold respectively in the HFD-fed mice. Genes related to central nervous system morphogenesis were significantly down-regulated, such as homeobox D11 (Hoxd11) (12.21-fold) and UNC homeobox (Uncx) (5.39-fold). Inflammatory/immune related processes were altered as well. The mRNA levels of chemokine (C-C motif) receptor 1(Ccr1) was up-regulated to 13.18-fold. The rest immune-related genes including chemokine (C-C motif) receptor 4 (Ccr4), CD200 receptor 3 (Cd200r3), CD274 molecule (Cd274), CD300 antigen like family member G (Cd300lg) and transcription factor AP-2, alpha (Tfap2a) were down-regulated to 8.28-,3.92-, 3.48-, 11.96- and 4.79-fold respectively.

**Table 3:** Differentially expressed genes in the brain of HFD-fed mice

Gene symbol	Log_2_FC	*P*- value	Gene symbol	Log_2_FC	*P*- value	Gene symbol	Log_2_FC	*P*- value
Alpi	−3.65	0.022	Slc22a19	−3.40	0.008	Gm13986	−2.48	0.019
Alpk3	−6.59	0.009	Sly	−6.59	0.009	Gm14302	−6.28	0.016
Bhlha15	−3.52	0.027	Smim24	−3.65	0.022	Gm14673	−2.43	0.018
Bmp8b	−1.82	0.039	Spata18	−5.87	0.048	Gm14886	−6.02	0.033
Brs3	2.80	0.034	Tfap2a	−2.26	0.033	Gm15174	−2.49	0.036
C6	−6.15	0.023	Tnfsf15	−5.87	0.048	Gm15302	−5.87	0.048
C87414	−5.87	0.048	Uncx	−2.43	0.041	Gm15839	−1.96	0.047
Capn11	−2.98	0.010	Usp17la	−1.86	0.039	Gm16028	−6.15	0.023
Ccr1	3.72	0.046	Vax2os	−2.15	0.017	Gm16060	−5.87	0.048
Ccr4	−3.05	0.008	Zfp345	−2.89	0.032	Gm16513	−5.87	0.048
Cd200r3	−1.97	0.040	1700007P06Rik	−6.02	0.033	Gm17085	−3.94	0.011
Cd274	−1.80	0.037	1700021A07Rik	−3.38	0.042	Gm17535	−7.89	5.53E-09
Cd300lg	−3.58	0.005	1700128F08Rik	−2.23	0.012	Gm20505	−2.04	0.046
Ces5a	−1.83	0.034	1810019N24Rik	−6.02	0.033	Gm20663	−1.98	0.044
Cpa4	−5.87	0.048	2810404F17Rik	−4.30	4.67E-04	Gm20831	−6.69	0.006
Csprs	−3.38	0.042	3110067C02Rik	−5.87	0.048	Gm21292	−3.39	0.003
Dsg3	−2.23	0.022	4931406B18Rik	−1.69	0.050	Gm2165	−2.71	0.020
Egfros	−6.39	0.016	9530056K15Rik	−5.87	0.048	Gm21719	−6.15	0.023
Esp6	−6.86	0.005	C230088H06Rik	−4.17	0.005	Gm21738	−6.61	2.23E-09
Ffar2	−3.38	0.042	C430042M11Rik	−3.06	0.002	Gm21776	−6.69	0.006
Gcm1	−2.38	0.013	G430049J08Rik	−2.29	0.025	Gm21784	−3.77	0.015
Hopxos	−5.87	0.048	CH36-246D16.4	−3.02	0.009	Gm21989	6.03	0.048
Hoxd11	−3.61	3.48E-04	CH36-35H7.2	−7.54	0.001	Gm23897	−2.55	0.009
Insrr	−2.45	0.010	CH36-399D20.1	−7.93	1.73E-04	Gm26573	−7.72	3.41E-04
Itgb6	−3.28	0.001	Gm10038	−2.16	0.026	Gm26583	−6.15	0.023
Klk14	−3.48	0.002	Gm10132	−6.02	0.033	Gm26648	−6.28	0.016
Lmx1b	−3.02	0.003	Gm10134	−1.96	0.036	Gm26704	−5.05	9.17E-06
Lypd8	−4.17	0.005	Gm10172	−2.31	0.013	Gm26705	−2.22	0.017
Mep1b	−2.76	0.020	Gm10715	−6.88	1.29E-09	Gm26719	−2.84	0.015
Mid1	−1.78	0.045	Gm10717	−6.11	3.59E-09	Gm26763	−6.15	0.023
Mpz	−5.87	0.048	Gm10718	−6.15	2.98E-09	Gm26804	−7.01	0.003
Ms4a4b	−7.01	0.003	Gm10719	−6.56	5.50E-10	Gm26857	−2.14	0.022
Muc19	−2.46	0.011	Gm10720	−4.94	1.06E-06	Gm26870	−6.03	4.96E-09
Muc6	−5.87	0.048	Gm10721	−8.04	1.26E-04	Gm27956	−6.39	0.016
Mylk4	−2.85	0.002	Gm10722	−5.19	7.91E-07	Gm3755	−1.96	0.036
Nkapl	−2.72	0.046	Gm10800	−6.03	4.95E-09	RP23-184B11.4	−6.28	0.016
Nkx2-1	−3.40	2.13E-04	Gm10801	−5.91	8.73E-09	RP23-315H12.7	−5.87	0.048
Orly	−6.69	0.006	Gm11168	−5.67	3.66E-08	RP23-458B6.16	−6.86	0.005
Otc	2.70	0.032	Gm11231	−2.18	0.032	RP23-91 L14.2	−6.59	0.009
Patl2	−5.87	0.048	Gm11398	−5.87	0.048	RP24-112B7.3	−3.24	0.012
Pcdh12	2.83	0.046	Gm11883	−5.87	0.048	RP24-209E1.3	−6.02	0.033
Pou1f1	−2.62	0.003	Gm11948	−5.87	0.048	RP24-228I22.1	−6.69	0.006
Pou2f2	−1.83	0.043	Gm12177	−2.43	0.021	RP24-319B23.2	−4.68	0.001
Pou2f3-rs1	−6.02	0.033	Gm12496	−2.91	0.013	RP24-369 J17.1	−6.39	0.016
Psg28	−1.83	0.042	Gm12652	−6.28	0.016	RP24-446E18.2	−6.02	0.033
Rbp2	−3.72	0.018	Gm13086	−2.34	0.023	RP24-482E11.1	−5.87	0.048
Rdh19	−6.28	0.016	Gm13465	−2.54	0.033	RP24-72B9.10	−6.02	0.033
Rhox8	−6.15	0.023	Gm13691	−6.28	0.016			
Serpina10	−4.52	0.002	Gm13961	−5.87	0.048			

*FC* fold change

### Genes and the related biological processes altered in the spleen of HFD-fed mice

61 genes were markedly changed in the spleen, however, half of which were largely unknown. The overrepresented biological processes in the spleen were mainly related to acute-phase response and immune system, with a significant change in the expression of immune-related genes (Fig. 3). As shown in Table 4, immunoglobulin kappa joining 4 (Igkj4) and T cell receptor alpha joining 37(Traj37) were increased to 8.34- and 70.52-fold respectively, while Fc receptor-like S, scavenger receptor (Fcrls), immunoglobulin heavy variable 1–84 (Ighv1-84), immunoglobulin kappa joining 1(Igkj1), immunoglobulin kappa variable 5–39 (Igkv5-39), regenerating islet-derived 3 alpha (Reg3a) and regenerating islet-derived 3 beta (Reg3b) in various immunological pathways were decreased to 74.54-, 11.63-, 54.57-, 3.60-, 16.34- and 8.11- fold respectively. Other overrepresented biological processes included carbohydrate metabolism, ubiquitin-dependent protein catabolism, G2/M transition of mitotic cell cycle and neurotransmitter uptake.

**Table 4:** Differentially expressed genes in the spleen of HFD-fed mice

Gene symbol	Log_2_FC	*P*- value	Gene symbol	Log_2_FC	*P*- value
Acr	−6.09	0.033	5330434G04Rik	−5.77	0.048
Ccdc38	−6.09	0.033	5730596B20Rik	−5.77	0.048
Cngb3	−5.77	0.048	AY036118	−6.09	0.033
Fau-ps2	2.73	0.027	BC061212	−3.01	0.046
Fcrls	−6.22	0.023	BC068157	5.92	0.048
Folr2	−6.22	0.023	D830025C05Rik	−5.94	0.033
Frmpd4	−6.35	0.016	Gm10071	−5.77	0.048
Hs3st5	−5.77	0.048	Gm11455	2.26	0.047
Ighv1-84	−3.54	2.38E-04	Gm11800	−3.62	0.010
Igkj1	−5.77	0.048	Gm11957	6.33	0.023
Igkj4	3.06	0.021	Gm12763	−2.40	0.034
Igkv5-39	−1.85	0.034	Gm13483	−3.01	0.046
Lnx1	−5.94	0.033	Gm14444	−6.09	0.033
Lrrc7	−2.83	0.046	Gm15302	−6.22	0.023
Mcpt4	−3.01	0.046	Gm15785	−5.94	0.033
mt-Tm	−5.77	0.048	Gm17305	−5.94	0.033
Nek10	−5.94	0.033	Gm20544	−3.01	0.046
Palm2Akap2	−3.21	0.027	Gm21719	−3.30	0.027
Pcdhb10	−6.09	0.033	Gm2237	−5.94	0.033
Reg2	−3.33	2.65E-04	Gm24436	6.33	0.023
Reg3a	−4.03	1.50E-04	Gm25153	2.80	0.035
Reg3b	−3.02	0.001	Gm25931	2.65	0.046
Rpl9-ps3	−5.94	0.033	Gm26619	−2.95	0.008
Slc17a6	−5.77	0.048	Gm26807	−6.09	0.033
St6gal2	−5.77	0.048	Gm26825	−2.37	0.029
Tceal3	−3.01	0.046	Gm6136	−6.22	0.023
Tpd52l1	−5.77	0.048	Gm6612	2.65	0.046
Traj37	6.14	0.033	RP23-446G23.1	2.80	0.035
Vat1l	−3.01	0.046	RP24-369 J17.1	−2.89	0.039
Zfp42	−5.77	0.048	RP24-44H8.4	−2.86	0.011
1700095A21Rik	−5.94	0.033			

*FC* fold change

### Genes and the related biological processes altered in the lung of HFD-fed mice

In the lung, 83 genes were significantly altered. The overrepresented biological processes were mainly enriched in immune-related processes, including T cell and B cell mediated immune response (Fig. 3). As shown in Table 5, Immunoglobulin heavy constant epsilon (Ighe) and immunoglobulin heavy constant gamma 1 (G1m marker) (Ighg1) were increased to 52.71- and 6.02-fold respectively, while the mRNA levels of chemokine (C-C motif) receptor 9 (Ccr9), CD8 antigen, alpha chain (Cd8a), recombination activating gene 1(Rag1), recombination activating gene 2 (Rag2) and suppression inducing transmembrane adaptor 1 (Sit1) were decreased to 6.32-, 3.94-, 512-, 19.70- and 5.10- fold respectively. Circulatory system process was also significantly changed in the lung of HFD-fed mice, with the down-regulation of natriuretic peptide precursor A (Nppa) (7.78-fold) and natriuretic peptide precursor B (Nppb) (21.26-fold). Leptin (Lep) involved in the regulation of cholesterol absorption were increased to 5.70-fold and CART prepropeptide (Cartpt) related to cell glucose homeostasis were decreased to 62.25-fold. Other overrepresented biological processes included circadian regulation of gene expression and positive regulation of transmission of nerve impulse.

**Table 5:** Differentially expressed genes in the lung of HFD-fed mice

Gene symbol	Log_2_FC	*P*- value	Gene symbol	Log_2_FC	*P*- value
Abcc6	5.72	0.048	Satb1	−1.99	0.022
Arpp21	−5.29	7.83E-07	Scn1a	−2.97	0.042
Arsi	−2.28	0.034	Sit1	−2.35	0.023
Bmp10	−4.72	4.94E-06	Skint10	−5.96	0.033
Cartpt	−5.96	0.033	Spo11	−3.46	0.001
Casp14	−5.96	0.033	St18	5.72	0.048
Ccr9	−2.66	0.004	Syt13	−2.12	0.039
Cd8a	−1.98	0.025	Syt2	−3.54	0.035
Cdca5	−2.28	0.036	Tdrd5	−2.93	0.006
Chrna9	−4.53	0.003	Tgm5	−2.78	0.039
Crisp1	−1.87	0.040	Themis	−2.26	0.010
Dntt	−7.36	2.18E-09	Tnni1	6.36	0.016
Elovl3	−1.96	0.036	Trbc1	−1.97	0.025
Epyc	−6.15	0.023	Trbv17	−6.32	0.016
Gucy2g	5.91	0.048	Trbv4	−3.66	0.027
Hist1h1a	−1.88	0.034	Trim10	1.90	0.040
Ighe	5.72	0.048	Tube1	−1.96	0.050
Ighg1	2.59	0.011	Ubd	−3.06	0.017
Ighv11-1	−2.90	0.002	Vmn2r96	−3.54	0.035
Ighv11-2	−3.20	0.001	Xkrx	−2.35	0.008
Ighv12-3	−3.46	0.001	Xlr5a	5.72	0.048
Ighv1-53	1.92	0.046	Zan	3.20	0.046
Ighv1-54	2.42	0.031	1600029O15Rik	5.91	0.048
Ighv1-84	−2.28	0.036	4930455G09Rik	−3.66	0.027
Ighv7-3	−2.04	0.041	9330132A10Rik	6.07	0.033
Ighv7-4	−6.47	0.012	BB031773	−7.46	0.001
Igkv14-126	−2.80	0.002	BC028471	−2.85	0.027
Igkv1-99	6.89	0.004	BC065403	−5.96	0.033
Igkv3-2	1.95	0.039	Gm10489	5.72	0.048
Igkv4-91	−2.95	0.002	Gm10715	5.91	0.048
Insc	5.91	0.048	Gm10717	1.74	0.045
Lctl	2.40	0.031	Gm10785	5.91	0.048
Lep	2.51	0.008	Gm13855	5.72	0.048
Lin28a	5.72	0.048	Gm15340	−6.47	0.012
Ltb4r1	2.93	0.022	Gm15405	−2.16	0.045
mt-Tt	6.22	0.023	Gm15576	−5.96	0.033
Nppa	−2.96	0.001	Gm20438	5.91	0.048
Nppb	−4.41	4.38E-05	Gm26202	6.07	0.033
Prom2	−2.66	0.031	Gm26316	5.72	0.048
Prss16	−9.54	4.44E-07	Gm26870	2.00	0.021
Rag1	−9.00	2.01E-12	RP23-230A14.1	5.72	0.048
Rag2	−4.30	3.9E-04			

*FC* fold change

### Verification of RNA-seq data

**Fig. 4:**
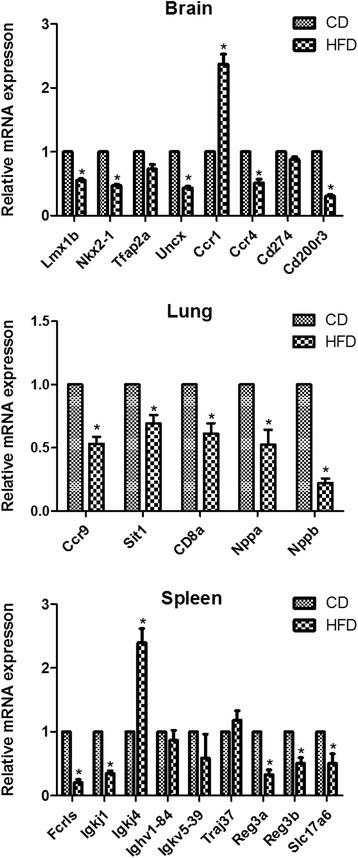
Confirmation of differential gene expression via qRT-PCR analysis (CD, control diet; HFD, high fat diet; *P < 0.05 vs. CD group). The expression value was normalized to the β-actin expression level. Values are expressed as average mRNA expression ± SEM bars

qRT-PCR was used to validate the expression levels measured by RNA-seq for 22 selected genes (8 genes in the brain, 9 genes in the spleen and 5 genes in the lung) from the list of differently expressed genes. As demonstrated in Fig. 4, qRT-PCR showed significant alterations in the expression of the 17 genes in correspondence with the findings from the RNA-sequencing analysis, while no obvious differential expression was detected for the 5 genes of Trap2a, Cd274, Ighv1-84, Igkv5-39 and Traj37 by qRT-PCR.

## Discussion

Consistent with previous reports [6, 16], our results showed that the consumption of a high-fat diet for 3 days significantly increased plasma glucose level. The high-fat diet also increased plasma cholesterol and HDL cholesterol concentrations. Plasma triglycerides concentration decreased significantly after short-term high-fat feeding. Indeed, decreased triglycerides level was previously reported as early as 3 days after beginning a high-fat diet in a study that involved mice fed a chronic high-fat diet [17]. A randomized, double-blind, crossover study in 12 healthy subjects reported that plasma triglycerides concentration was significantly lower after a 3-d high-fat diet [18]. The low plasma triglycerides could be due to increased liver triglyceride content, possibly resulting in triglycerides being stored in the liver [19].

To our knowledge, we first reported that 3 days of high-fat feeding can induce exercise performance decrease in mice prior to substantial weight gain. In contrast to previous studies which conducted microarray analysis in a single tissue [6–9], we used RNA-seq to investigate the global gene expression profiles of ten tissues in the early response to fat intake. Interestingly, our study showed that differential gene expression patterns existed in the ten tissues. Previous researches on the effect of HFD mainly focused on the liver, skeletal muscle, intestine and heart, which were thought to have significant responses to consumption of high fat diets. However, in our results, the brain, spleen and lung were demonstrated to have more pronounced transcriptional changes than other tissues following 3 days of high-fat feeding. The simultaneous analysis of multi-tissues by RNA sequencing yielded information which had not been revealed by previous analyses of a single tissue.

In the brain of HFD-fed mice, dopamine neurons differentiation related genes including Lmx1b, Nkx2-1 and Uncx were found to be significantly down-regulated. Lmx1b, a key transcription factor for the specification of dopaminergic cell fate, has been reported to increase midbrain size and allocation of dopamine progenitors by promoting Wnt1/Wnt signaling [20]. Deficiency of *Nkx2-1* in mice would lead to a remarkable abnormality in the trajectory of the ascending dopamine pathway [21]. Uncx, also known as Uncx4.1, is involved in the development of midbrain dopaminergic neurons [22]. Therefore, the down-regulation of these genes may contribute to impaired exercise capacity, which is supported by the observation of a link between dopamine and levels of physical exercise [23]. Moreover, Lmx1b and Nkx2-1 were demonstrated to regulate the migration of the superficial dorsal horn neurons and interneurons to the striatum or cortex, respectively [24, 25]. The decreased expression of the two genes suggested disturbed neuronal migration in HFD-fed mice, which may result in abnormal development of the nervous system. Inflammation is considered to be one of the important factors for deterioration of physical performance [26]. Similarly, our results also suggested that many immune-related genes were significantly altered in the brain of HFD-fed mice. Ccr1, which involved in the host response to pathogens and several inflammatory conditions [27], were significantly up-regulated. On the other hand, Ccr4 and Cd200r3 showed decreased expression in HFD-fed mice. Ccr4 were found to be functionally expressed on peripheral blood CD4^+^CD25^+^ regulatory T (Treg) cells [28]. CD200 imparts an immunoregulatory signal through the receptor for CD200, leading to the suppression of T-cell–mediated immune responses [29]. The up-regulation of Ccr1, coupled with the down-regulation of Ccr4 and Cd200r3, suggested the up-regulation of an inflammatory response toward the high fat diet in the brain. Consistently, it was reported that consumption of a HFD with 1 to 3 days induced hypothalamic inflammatory in both rats and mice [30]. However, the genes related to hypothalamic inflammatory signaling in the above study were not found to be significantly altered in our results. This discrepancy may reflect a difference between specific brain region and the whole brain.

The immune system has been considered to be affected by HFD exposure over a period of several weeks [31]. However, in the present study, we found that 3 days of HFD feeding had induced disturbed immune response in spleen, which is a major organ involved in B-cell maturation. Fcrls, which belongs to Fc receptor-like family possessing inhibitory and/or activating signaling motifs in B cell differentiation [32], were significantly down-regulated in response to HFD feeding. Similarly, microarray profiling carried out by Cui et al. in the spleen of C57BL/6 mice fed on a HFD also showed a decreased expression of Fc receptor [33]. Meanwhile, we observed abnormal expression of a few genes involved in immunoglobulin/B cell receptor signaling. Igkj4 showed significant up-regulation in HFD-fed mice, whereas the expression of Igkj1 were significantly decreased. Additionally, Reg3a and Reg3b, which play critical roles in acute-phase response [34, 35], were significantly down-regulated. The findings suggested that immune dysfunction was implicated in the spleen in response to 3 days of HFD feeding. Interestingly, neurotransmitter uptake related genes were significantly changed, such as solute carrier family 17 (sodium-dependent inorganic phosphate cotransporter), member 6(Slc17a6) which plays a key role in the transport of glutamate into synaptic vesicles before exocytotic release and the regulation of glutamate signaling [36].

Evidence has increasingly shown that a HFD regarded as a primary cause for numerous diseases including diabetes, hypertension, and steatohepatitis. However, few studies have been carried out to examine the effect of a HFD on the lung. To our surprise, the PCA results showed pronounced transcriptional changes in the lung and this is the first report to investigate the lung transcriptome profile after 3 days of HFD feeding. As the lung is a major site of immune regulation, our results revealed that many immune-related genes were significantly altered. Chemokine receptor Ccr9, which have proved to be important in the Treg cells mediated self-tolerance [37], was markedly down-regulated in HFD-fed mice. Sit1, a critical negative regulator of TCR-mediated signaling [38], showed significant down-regulation as well. The decreased expression of Ccr9 and Sit1 suggested an activated inflammatory response affected by HFD in the lung, which was in agreement with the previous study of the involvement of a HFD on lung inflammation [39]. In addition, we discovered decreased expression of CD8a in HFD-fed mice, which is important in cell-mediated immune defense and T-cell development [40]. Moreover, genes involved in T cell receptor signaling and immunoglobulin/ B cell receptor signaling were found to be significant altered. This study also revealed that circulatory system related genes were down-regulated by HFD in the lung. Nppa and Nppb are the precursor of atrial natriuretic peptide (ANP) and b-type natriuretic peptide (BNP), which have important physiological functions in the regulation of vascular tone and plasma volume [41]. ANP exhibits a protective role in the lung function in acute lung injury apart from its vasodilatory and natriuretic effects [42]. Intake of a HFD had been proved to make a slower pulmonary O_2_ uptake kinetics and attenuate microvascular blood flow and O_2_ delivery during the transition to moderate intensity exercise [43]. Therefore, we speculated that the decreased expression of Nppa and Nppb may contribute to the impaired exercise capacity in HFD-fed mice.

## Conclusions

The simultaneous analysis of ten tissues following 3 days of high-fat feeding by RNA-seq technology revealed that the brain, spleen and lung had more pronounced transcriptional changes than other tissues. Dysregulation of peripheral and central immune response had been implicated in the early stage of the response to HFD exposure. Neurotransmission-related genes and circulatory system process related genes were markedly down-regulated in the brain and lung, respectively. These findings provide new insights for the deleterious effects of a HFD and contribute to the understanding of molecular mechanisms of exercise performance decrease induced by short-term high-fat feeding.

## Additional files

Additional file 1: Table S1. List of Primer sequences used for qRT-PCR. (DOC 57 kb)
Additional file 2: Table S2. Plasma biochemical parameters of CD-fed and HFD-fed mice. (DOCX 21 kb)

## Abbreviations

ALB: Albumin
ALP: Alkaline phosphatase
ALT: Alanine aminotransferase
ANP: Atrial natriuretic peptide
ApoE: Apolipoprotein E
AST: Aspartate aminotransferase
BNP: b-type natriuretic peptide
Ca: Calcium
CD: Control diet
CK: Creatine kinase
Cl: Chlorine
CPM: Counts per million
Cr: Creatinine
CRP: C-reactive protein
CysC: Cystatin C
DBIL: Direct bilirubin
DEGs: Differentially expressed genes
HBDH: A-hydroxybutyric acid dehydrogenase
HCY: Homocysteine
HDL-C: High-density lipoprotein-cholesterol
HFD: High fat diet
IBIL: Indirect bilirubin
K: Potassium
LDH: Lactate dehydrogenas
LDL-C: Low-density lipoprotein-cholesterol
MAS: Molecule annotation system
Na: Sodium
PCA: Principle component analysis
RNA-seq: RNA sequencing
RPKM: Reads Per Kilobase of exon model per Million mapped reads
SOD: Superoxide dismutase
TBA: Total bile acid
TBIL: Total bilirubin
TP: Total protein
Treg: Regulatory T
UA: Uric acid
