# Supplementary material for: The effects of short-term high-fat feeding on exercise capacity: multi-tissue transcriptome changes by RNA sequencing analysis
Source: Lipids Health Dis. 2017 Feb 2;16:28. doi: 10.1186/s12944-017-0424-7 (PMC5290644; doi:10.1186/s12944-017-0424-7)
Supplement: Additional file 1: Table S1. — List of Primer sequences used for qRT-PCR. (DOC 57 kb) [file 12944_2017_424_MOESM1_ESM.doc]

| Gene symbol | Sequences (5’-3’) | | Annealing Temperature (^o^C) | Product length (bp) |
| --- | --- | --- | --- | --- |
|  | Forward primer (5'->3') | Reverse primer (5'->3') |  |  |
| Lmx1b | CTGTGAAGAGTGAGGATGAAG | CTGTGTGGTGAGGATGGT | 60 | 131 |
| Nkx2-1 | CTTCTGTTTCCTTCTTCCTTC | TGTTCTCTGTCCTTCTCTGTT | 60 | 155 |
| Tfap2a | TGCTTTGGTTATTGTTGTTG | GCTGGTGTAGGGAGATTG | 60 | 177 |
| Uncx | CGAGTTCAGGTCTGGTTC | GTGTTTGCGTTTCTTCTTCT | 60 | 180 |
| Ccr1 | CTTCTATTCTTCCTCCTCTGG | GTTGCTTACTCTGCTCACAC | 60 | 100 |
| Ccr4 | CAAGGAAGGTATCAAGGCA | CAGGACGAACAGCAAATC | 60 | 181 |
| CD274 | GCAACACATCCTCCACAG | GCCACATTTCTCCACATC | 60 | 132 |
| Cd200r3 | GAGTGAGGTTGTGCTGGA | GCTGTGGATAAAATGCTTG | 60 | 141 |
| Fcrls | CTTGCTTGTTCTGTGGCT | ACGCTGACTTTTCTTCCC | 60 | 93 |
| Igkj4 | AAAGAAGACAGCCACAAGAA | CGGACACAACACAGAATG | 60 | 177 |
| Ighv1-84 | GAGTGGATTGGATGGATTT | CTGTGCTGGAGGATGTGT | 60 | 100 |
| Igkj1 | CCGTTGTCTATGTCTGTGG | TCTGTATCTTTGCCTTGGAG | 60 | 95 |
| Igkv5-39 | GCTTCCCAATCCATCTCT | ACATCTTCAGGTTCCACACT | 60 | 98 |
| Traj37 | TTTGGACTGGGGACAACT | AAGAAAGGAAAGCCTGAAAC | 60 | 142 |
| Reg3a | AAGCAGTAGACAAGCAGCA | GATGGATGAAAGAGGACAGA | 60 | 144 |
| Reg3b | GCTTCATTCTTGTCCTCCA | TTACTCCATTCCCATCCAC | 60 | 119 |
| Slc17a6 | GCGATACTGCTCACCTCTAC | CCAACCTACTCCTCTCCAA | 60 | 172 |
| Ccr9 | AATCTCTGGTCTGCCTTTG | GGCTTGTGAGTTCTGTGG | 60 | 104 |
| Sit1 | CACACCTTTCCCTCCATT | TATCCACCCACATCCTCTC | 60 | 139 |
| CD8a | GATGCTCTTGGCTCTTCC | CTTCGTTTTCCTTGCTGA | 60 | 189 |
| Nppa | CAAGGAGGAAAAGGCAGT | GTGATAGATGAAGGCAGGAA | 60 | 145 |
| Nppb | CGAGACAAGGGAGAACAC | AAGGAAAAGCAGAAACAGAA | 60 | 101 |
| β-actin | GGGAAATCGTGCGTGAC | AGGCTGGAAAAGAGCCT | 60 | 176 |

**Table S1.** **List of Primer sequences used for qRT-PCR**
