# Supplementary material for: The effects of short-term high-fat feeding on exercise capacity: multi-tissue transcriptome changes by RNA sequencing analysis
Source: Lipids Health Dis. 2017 Feb 2;16:28. doi: 10.1186/s12944-017-0424-7 (PMC5290644; doi:10.1186/s12944-017-0424-7)
Supplement: Additional file 2: Table S2. — Plasma biochemical parameters of CD-fed and HFD-fed mice. (DOCX 21 kb) [file 12944_2017_424_MOESM2_ESM.docx]

**Table S2.** Plasma biochemical parameters of CD-fed and HFD-fed mice

| Parameter | CD | HFD | *P* value |
| --- | --- | --- | --- |
| C-reactive protein (mg/L) | 0.26±0.02 | 0.20±0.02 | 0.131 |
| [Superoxide](javascript:void(0);) [dismutase](javascript:void(0);) (U/L) | 615.00±33.18 | 545.17±28.13 | 0.140 |
| Homocysteine (mmol/L) | 6.36±0.31 | 6.63±0.21 | 0.479 |
| Alanine aminotransferase (U/L) | 7.00±0.54 | 4.83±2.40 | 0.102 |
| Aspartate aminotransferase (U/L) | 74.40±5.00 | 71.00±5.91 | 0.679 |
| Alkaline phosphatase (U/L) | 57.00±2.12 | 55.67±2.17 | 0.717 |
| Total protein (g/L) | 48.66±0.49 | 48.63±0.64 | 0.915 |
| Albumin (g/L) | 28.40±0.38 | 28.41±0.40 | 0.977 |
| Globulin (g/L) | 20.26±0.39 | 20.22±0.36 | 0.936 |
| Albumin / Globulin | 1.40±0.04 | 1.41±0.02 | 0.947 |
| Total bilirubin (umol/L) | 0.50±0.19 | 0.45±0.03 | 0.779 |
| Direct bilirubin (umol/L) | 0.18±0.13 | 0.18±0.07 | 0.982 |
| Total bile acid (umol/L) | 1.38±0.34 | 1.90±0.34 | 0.319 |
| Uric acid (umol/L) | 156.60±20.78 | 147.50±13.71 | 0.715 |
| Creatinine (umol/L) | 7.40±0.40 | 7.67±0.21 | 0.550 |
| Urea (mmol/L) | 9.95±0.73 | 10.00±0.48 | 0.948 |
| Cystatin C (mg/L) | 0.02±0.00 | 0.01±0.00 | 0.348 |
| Creatine kinase (U/L) | 238.00±37.54 | 119.00±14.70 | 0.011 |
| Lactate dehydrogenase (U/L) | 520.80±64.37 | 471.83±53.25 | 0.568 |
| A-hydroxybutyric acid dehydrogenase (U/L) | 198.00±23.45 | 182.67±17.89 | 0.609 |
| Potassium (K) (mmol/L) | 6.70±0.19 | 6.69±0.32 | 0.984 |
| Sodium (Na) (mmol/L) | 148.22±1.08 | 152.17±0.26 | 0.004 |
| Chlorine (Cl) (mmol/L) | 107.42±1.16 | 111.13±0.43 | 0.010 |
| Calcium (Ca) (mmol/L) | 2.07±0.02 | 2.04±0.02 | 0.467 |

Values are expressed as means± SEM. CD, control diet; HFD, high fat diet.
